# Effect of Portland Cement on the Selected Properties of Flue Gas Desulfurization Gypsum-Based Plasters

**DOI:** 10.3390/ma16145058

**Published:** 2023-07-18

**Authors:** Edyta Baran, Mariusz Hynowski, Łukasz Kotwica, Jacek Rogowski

**Affiliations:** 1 Research and Development Center, Branch Leszcze, Atlas sp. z o.o., Leszcze 15, 28-400 Pińczów, Poland; mhynowski@atlas.com.pl; 2 Department of Building Materials Technology, Faculty of Materials Science and Ceramics, AGH University of Science and Technology, al. Adama Mickiewicza 30, 30-059 Kraków, Poland; lkotwica@agh.edu.pl; 3 Institute of General and Ecological Chemistry, Łódź University of Technology, ul. Żeromskiego 116, 90-924 Łódź, Poland; jacek.rogowski@p.lodz.pl

**Keywords:** gypsum–cement mortars, thaumasite, ettringite, expansion, mechanical strength

## Abstract

The introduction of the European Union’s climate change legislation and the intended use of renewable energy sources instead of fossil fuels will significantly reduce the production of flue gas desulfurization (FGD) gypsum used as the raw material for gypsum mortar plasters’ production. This has forced mortar producers to look for alternative materials, including gypsum–cement composites. This work investigated the mechanical strength and linear extension of four gypsum–cement mortars with the gypsum content reduced to 30%. The authors showed that the cement admixture of 6 to 12% introduced into the prepared mortars resulted in the formation of gypsum–cement mortars, which fulfill the requirements of the EN 13279-1:2008 standard concerning mechanical strength. This publication took into account the use of scanning electron microscopy (SEM), energy-dispersive X-ray spectroscopy (EDS), and X-ray diffractometry to characterize the chemical and phase composition of the mortars up to 180 days of dry air curing and increased relative humidity (RH) conditions. The formation of thaumasite, ettringite, and mixed ettringite–thaumasite phases was interesting because of their deleterious effect on the durability of plaster mortars.

## 1. Introduction

Gypsum plasters are among Poland’s most-widely used finishing materials for walls inside buildings. Gypsum binder, which is the main binder used in these materials, can be obtained by the calcination of calcium sulfate dihydrate mined from natural deposits or as a by-product from the flue gas desulfurization (FGD) process of sulfur dioxide removal from flue gases emitted by heat and power plants. The total amount of gypsum binder obtained by the FGD method and from natural sources was 445,000 t in 2000 and increased considerably to 1,851,000 t in 2021 [1]. A significant amount of gypsum obtained in Poland from 2011 to 2020, equal to 37%, was used to produce gypsum mortars. The remaining part of gypsum was used for the production of mortar drywalls (36%), as a raw material in the cement industry (22%), and for other applications (5%) [2].

The primary source of naturally occurring gypsum in Poland is rich Miocene and Upper Permian (Zechstein) deposits mined, respectively, near the city of Pińczów (Świętokrzyskie voivodeship) and the city of Bolesławiec (Lower Silesia). Miocene deposits are non-homogeneous and contain only 85 to 92% calcium sulfate dihydrate due to notable inclusions such as clays, limestone, or silica. The Zechstein calcium sulfate deposits possess significantly higher purity, above 95%. However, they are more expensive to exploit than the Miocene deposits because they are placed at a greater depth and contain an admixture of anhydrite [3]. Although the flue gas desulfurization (FGD) process has become an additional source of gypsum in Poland since 1994, a significant increase in synthetic gypsum (FGD gypsum) production has occurred only since 2008 with the introduction of modern technologies, which allow reducing SO_2_ emission into the atmosphere [4].

Compared to natural gypsum, the vital advantage of FGD gypsum is its high calcium sulfate content, exceeding 95%. As a result, plaster mortars produced using synthetic gypsum have up to 40% higher mechanical strength than those obtained using natural gypsum [5]. In addition, FGD gypsum does not contain clay minerals. Hence, plaster mortars produced from it have high stability and homogeneity and are easy to apply [6]. These advantages, as well as the low cost and availability, have caused a substantial increase in the use of synthetic gypsum as a component of plaster mortars and other construction materials. The gypsum binder used for the gypsum plasters’ production is calcium sulfate hemihydrate (also called building gypsum) obtained in the process of the dehydration of the gypsum raw material (FGD gypsum or natural gypsum). The binder obtained in this way is mixed with water and then hydrates to form gypsum (calcium sulfate dihydrate). The hydration reaction of building-gypsum-based plasters is:

$${\mathrm{CaSO}}_{4}\cdot 0.5H_{2}O\;+1.5H_{2}O\;\to {\mathrm{CaSO}}_{4}\cdot 2H_{2}O$$

The basic regulation determining gypsum mortar types, compositions, and technical performance is the EN 13279-1:2008 standard [7]. This standard specifies seven types of gypsum plasters (B1–B7) and six kinds of gypsum plasters and mortars for special applications (C1–C7). Among these products, the most-widely produced in Poland and Europe are gypsum building plaster (B1), lightweight gypsum building plaster (B4), gypsum plaster with enhanced surface hardness (B7), and thin coat plaster (C6). According to this standard, mortar plasters of Types B1, B4, and C6 should have a flexural strength greater than 1.0 N/mm^2^ and a compressive strength above 2.0 N/mm^2^, whereas higher values of these parameters, no less than 2.0 N/mm^2^ and 6.0 N/mm^2^, respectively, are required for B7 gypsum plaster. In addition, the adhesion strength to the substrate for gypsum plasters should be no less than 0.10 N/mm^2^. All these mortar plasters should contain no less than 50% gypsum binder and comply with the EN 13279-1:2008 standard’s requirements. Typically, however, the lightweight and regular mortar plasters have a higher amount of gypsum binder (from 55 to 65%), which enables them to meet the standard’s requirements concerning mechanical parameters and allows for their efficient and easy use during application. In addition, the high content of gypsum binder results in a low density of 0.8 kg/m^3^ for lightweight gypsum plasters and 1.0 kg/m^3^ for gypsum plasters with enhanced surface hardness.

For a long time, a large amount of synthetic gypsum used as a binder in gypsum mortars produced in Poland was obtained by desulfurizing flue gases from burned coal in power plants.

This situation will change due to the implementation by the Polish government of the National Energy and Climate Plan for the years 2021–2030 [8], aimed at reducing the amount of coal in electricity generation to 56–60%. According to this regulation, fossil fuels used for electricity production will be replaced by renewable energy sources, resulting in the termination of FGD gypsum production by 2049 [9,10].

Consequently, the construction industry is trying to find new solutions related to replacing gypsum with other raw materials and increasing the use of natural gypsum, whose consumption has been declining since 1996. The development of modified materials is a long process involving manufacturing and application technology changes. Therefore, it seems helpful to introduce a temporary solution consisting of a new mortar technology that reduces the consumption of synthetic gypsum. Currently, the reduction of gypsum content below 50% is possible in plaster mortars of Types B2 (gypsum plaster) and B5 (lightweight gypsum plaster) as specified by EN 13279-1:2008 [7]. However, reducing the gypsum content in mortars can be challenging for manufacturers to obtain the required mechanical parameters while maintaining the current standards of application, quality, and performance. One possible solution to this problem includes adding Portland cement (PC) to the gypsum mortar. One can expect that cement addition will reduce the amount of gypsum in the mortar and improve its technical parameters.

Early attempts to use a mixture of gypsum and cement as a building material date back to the 1940s and 1950s. However, these attempts concerned applying gypsum–cement–pozzolana and gypsum–slag–cement–pozzolana composite materials to obtain construction materials [11]. In addition, there has been only a small number of papers published later on gypsum–cement composite materials containing gypsum as a binder, and they are mostly related to the improvement of gypsum’s properties such as waterproofing qualities, frost resistance [12,13,14,15,16,17], as well as its possible expanded use as a construction material [18].

The cement admixture significantly affects the properties of gypsum-based mortars, including the formation of new phases. For example, the hydration of cement in the presence of an excessive amount of ${\mathrm{SO}}_{4}^{2-}$ and ${\mathrm{CO}}_{3}^{2-}$ ions involves the appearance of the hydrated phases such as ettringite $\left({\mathrm{Ca}}_{6}{\mathrm{Al}}_{2}{\left[{\left(\mathrm{OH}\right)}_{4}\left({\mathrm{SO}}_{4}\right)\right]}_{3}\cdot 26H_{2}O\right)$ [19] and thaumasite $\left({\mathrm{Ca}}_{6}{\left[\mathrm{Si}{\left(\mathrm{OH}\right)}_{6}\right]}_{2}{\left({\mathrm{CO}}_{3}\right)}_{2}{\left({\mathrm{SO}}_{4}\right)}_{2}\cdot 24H_{2}O\right)$ [20,21,22]. Ettringite can positively affect the strength of gypsum–cement composites if formed before gypsum–cement paste loses its plasticity. Otherwise, the internal stress caused by ettringite expansion during its crystallization leads to gypsum–cement binder destruction [23]. In contrast to ettringite, thaumasite is soft, with no cohesion phase, whose formation leads to the breakdown of the C-S-H structure [24]. The crystal structures of thaumasite and ettringite are similar, and therefore, both phases can form solid solutions called woodfordite phases [25,26,27]. The formation of thaumasite and delayed ettringite induces internal stress on the walls of pores, which increases small pores [28,29,30,31,32]. Studies have shown that the volumetric expansion of thaumasite formed by the woodfordite route is much lower than the expansive capacity of ettringite. Despite this, thaumasite in gypsum–cement mortars can have unfavorable effects on their mechanical properties [33,34]. Various factors influence the formation of the crystalline phases of ettringite and thaumasite crystalline phases [35,36,37]. Gawlicki and Mróz studied thaumasite formation using gypsum, Na_2_SO_4_, and K_2_SO_4_ as the source of ${\mathrm{SO}}_{4}^{2-}$ ions, and they showed that the rate of thaumasite formation and its final amount were highest when gypsum was the source of the sulfate ions [38]. Other studies were devoted to applying silica fume, fly ash, blast furnace slag, and metakaolin as a corrosion-inhibiting admixture protecting gypsum–cement and cement mortars against thaumasite formation [39,40,41,42,43,44,45].

The object of this study was light gypsum–cement mortars containing 30% gypsum and different amounts of Portland cement (PC). The gypsum content of 30% was almost two-times less than typically used, so a Portland cement admixture of 6 to 12% was introduced into plaster mortars to obtain their mechanical parameters, which satisfied the standard’s requirements. The presented study comprised compressive and flexural strength measurements and linear expansion of gypsum–cement mortars as a function of curing time at controlled humidity. The curing time was increased to 180 days to analyze the unfavorable effect of ettringite and thaumasite formation on the durability of the plaster mortars. At the same time, tests were carried out in dry air conditions. Scanning electron microscopy (SEM), energy-dispersive X-ray spectroscopy (EDS), and X-ray diffractometry were used to characterize the microstructure of the mortars.

## 2. Materials and Methods

### 2.1. Materials

In this work, four types of gypsum–cement plaster were studied. Every kind of plaster contained 30% gypsum binder (d50 = 37.78 mm) obtained from FGD gypsum (Kozienice Power Plant) and different amounts (6, 8, 10, and 12%) of Portland cement CEM I 42.5R (19.94% SiO_2_, 5.14% Al_2_O_3_, 2.89% Fe_2_O_3_, 63.63% CaO, 1.4 % MgO, 3.17% SO_3_, 0.6% K_2_O, 0.2% Na_2_O, 0.074% ${\mathrm{Cl}}^{-}$, 0.12% P_2_O_5_) produced by Lafarge Kujawy.

Table 1 presents the composition of the prepared gypsum–cement plasters. They are named according to the scheme XG/YC, where X is equal to the gypsum content in mortars (30%) and Y for the cement fraction (6, 8, 10, and 12%).

Quartz sand, limestone powder, and expanded perlite were used as fillers. Hydrated lime, modified methyl cellulose (viscosity 35,000 mPa·s), and starch ether (viscosity 1000 mPa·s) were added to improve the plasticity and workability of the mortar. In addition, sodium lauryl sulfate as an air-entraining admixture and L (+) tartaric acid as a setting-time regulator were used.

### 2.2. Methods

#### 2.2.1. Gypsum–Cement Mortar Samples

The consistency of the mortars was measured using a flow table test with a (160 ± 5) mm spread diameter following the PN EN 13279-2:2014 standard [46]. Subsequently, the mortar samples were prepared as (4 × 4 × 16) cm beams.

The gypsum–cement mortar samples were stored for 180 days at two relative humidity (RH) conditions—increased RH (>95%) and a dry air environment (65 ± 5)% RH at temperatures from 18 °C to 20 °C. After seven days of storage at increased RH, a part of the samples was further conditioned at normal humidity conditions (65 ± 5)% RH. The mechanical strength and analysis by the XRD, EDS, and SEM methods were measured for samples dried to a constant mass at a temperature of 45 °C.

#### 2.2.2. Analytical Methods

##### Mechanical Strength

Flexural and compressive strength measurements were performed following the PN-EN 13279-2:2014 standard [46]. The samples conditioned for 2, 7, 28, and 180 days were tested.

##### Dimension Changes

Linear expansion tests were performed according to the PN-85 B-04500 [47] standard using the length comparator (Graff-Kaufman apparatus) with a measurement accuracy of 0.01 mm. The tests were carried out on prismatic samples of dimensions 4 × 4 × 16 cm with steel gauge studs attached to the square and front walls of the samples. The result of the determination was the arithmetic mean of three measurement results for each of the mortars. Samples were tested 24 h after mixing and up to 6 months, as shown in the result Section 3.2.

##### XRD Analysis

Powder X-ray diffraction analysis was performed using a Philips PW 1030 diffractometer operating with CuKα radiation. XRD data were collected in the range of 5—65° 2θ with a step of 0.05° and an exposition per one step of 1 s. The XRD patterns were collected for gypsum–cement mortar samples cured for 2, 7, 14, 28, and 180 days at increased RH (>95%). 

##### Scanning Electron Microscopy (SEM) and Energy Dispersive X-ray Spectroscopy (EDS)

The scanning electron microscope FEI NanoSEM in low vacuum mode of operation (residual water vapor pressure of 60 Pa) was used to acquire secondary electron (SE) images of four series of samples after 7 and 180 days of curing. Energy-dispersive X-ray spectroscopy was applied to study the sample’s elemental composition.

## 3. Results and Discussion

### 3.1. Mechanical Strength

**Figure 1:**
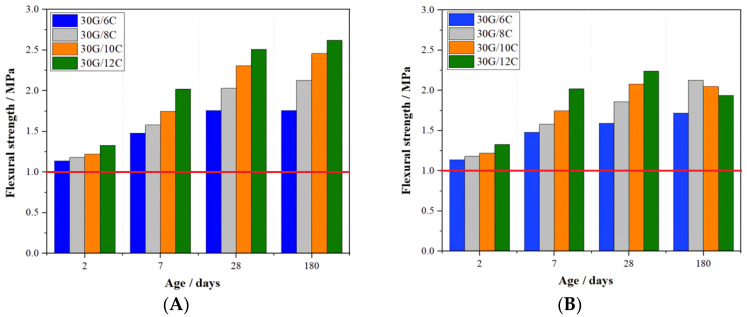
Flexural strength of gypsum–cement mortars (**A**) cured at increased relative humidity (>95%) and (**B**) in dry air conditions; red lines indicate the minimal required flexural strength (1 MPa) according to EN 13279-1:2008.

**Figure 2:**
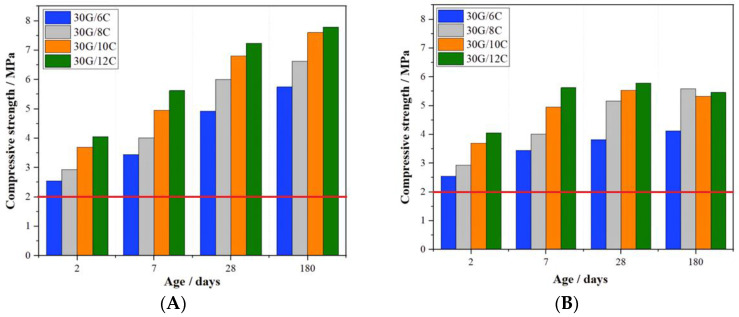
Compressive strength of gypsum–cement mortars (**A**) cured at increased relative humidity (>95%) and (**B**) in dry air conditions; red lines indicate the minimal required compressive strength (2 MPa) according to EN 13278-1:2008.

Figure 1 and Figure 2 present the results of the gypsum–cement mortars’ flexural and compressive strength measurements.

Figure 1A and Figure 2A clearly show that the flexural and compressive strengths of the gypsum–cement mortars cured in high relative humidity conditions (>95%) increased with the curing time and the cement content. It was found that the increase in the cement content of the plasters from 6 to 12% resulted in an increase in the 2-day flexural and compressive strength by 14.7 and 37.2%, respectively. The favorable effect of increased cement content on the mechanical strength of the plaster was also observed after 180 days of curing, as the flexural and compressive strength of the plaster containing 12 % were 32.7 and 26.1% higher than those for plaster with a cement content of 6%.

Notably, plaster mortar containing even as low as a 6% addition of Portland cement satisfied the standard’s requirements [7] for flexural and compressive strength as early as after two days of curing.

The growth of the mechanical strength was also observed after 180 days of conditioning for mortars initially stored for 7 days at increased relative humidity and then in dry air conditions (Figure 1B and Figure 2B). In this case, however, there were slight decreases in the flexural strength (30G/12C) and compressive strength (30G/10C and 30G/12C) after 180 days of curing relative to those strengths measured after 28 days of curing. Nevertheless, these mortars’ flexural and compressive strength after 180 days of curing in dry air conditions complied with the EN 13279-1:2008 standard [8]. Differences in the mechanical strength values obtained for mortars stored in the two different conditions can be explained by the lower content of the C-S-H phase in the plasters cured in dry air conditions.

It is important to note that the tested mortars’ bulk densities in their dry state ranged from 1038.7 kg/m^3^ to 1075.2 kg/m^3^.

### 3.2. Dimension Changes

**Figure 3:**
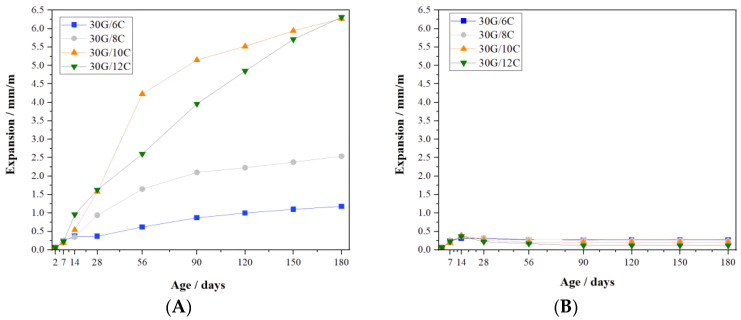
Dimension changes of gypsum–cement mortars (**A**) cured at increased relative humidity (>95%) and (**B**) in dry air conditions.

Figure 3 presents the dimension changes of the gypsum–cement mortars.

The expansions of all series of gypsum–cement mortars showed similar values for the initial 7 days of curing (Figure 3A,B). After that time, significant differences in the expansion of gypsum–cement mortars with different cement content were observed upon prolonged curing at increased relative humidity (Figure 3A). It was found that the rate of expansion of the mortars containing 6 and 8% cement decreased after 90 days of curing relative to other samples. Finally, the expansion of the 30G/6C and 30G/8C samples was about six- and three-times lower than those of the 30G/10C and 30G/12C samples. The expansion values after 180 days of curing at increased RH (>95%) are given in Table 2.

Each type of gypsum–cement mortar after 14 days in dry air conditions showed small expansion followed by the shrinkage of the gypsum–cement mix (Figure 3B). There was no significant expansion or shrinkage of the samples between 28 and 180 days of conditioning. The presented results suggest that thaumasite and ettringite were the main phases that influenced the observed mortar’s length changes, as observed for the studied samples.

### 3.3. X-ray Diffractometry

The gypsum–cement mortars cured for 2, 7, 14, 28, and 180 days at increased RH (>95%) were analyzed by X-ray diffractometry. The XRD patterns indicated that the analyzed samples contained some crystalline phases such as calcite, portlandite, quartz, thaumasite (JCPDS Card No. 01-072-2148), and ettringite (JCPDS Card No. 01-072-0646). No peaks characteristic of the C-S-H phase were present in the XRD patterns, which can be accounted for by the low crystallinity of this phase [48], the relatively low cement content in the mortars, and the possible overlap of the C-S-H phase diffraction peaks with those of gypsum and calcite.

The XRD analysis revealed the presence of unreacted calcium sulfate hemihydrate in the gypsum–cement samples containing 6, 8, and 10% cement after 2 days of curing. In contrast, no bassanite peaks were visible in the XRD patterns of the mortar containing 12% Portland cement after just two days of curing. It was found that calcium sulfate hemihydrate in the plaster having 6% cement did not react entirely after 7 days of curing. An intermediate rate of the bassanite reaction was observed for mortars containing 8 and 10% Portland cement, for which the XRD peaks of bassanite were not observed for a curing time longer than two days.

**Figure 4:**
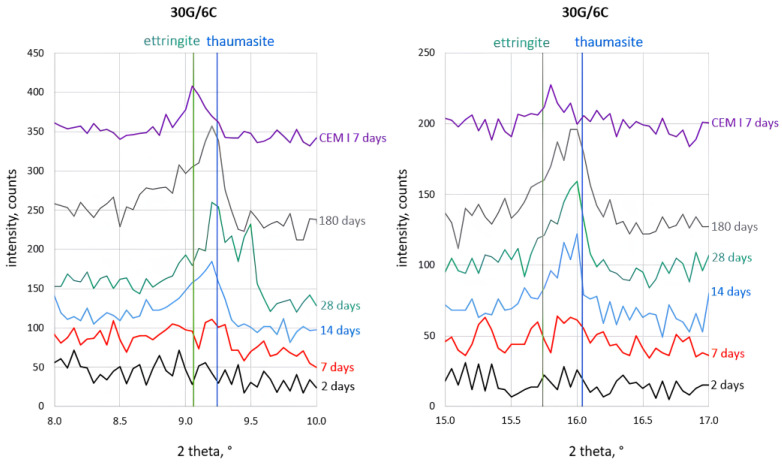
X-ray diffraction patterns of the 30G/6C sample cured at increased RH for 2, 7, 14, 28, and 180 days.

**Figure 5:**
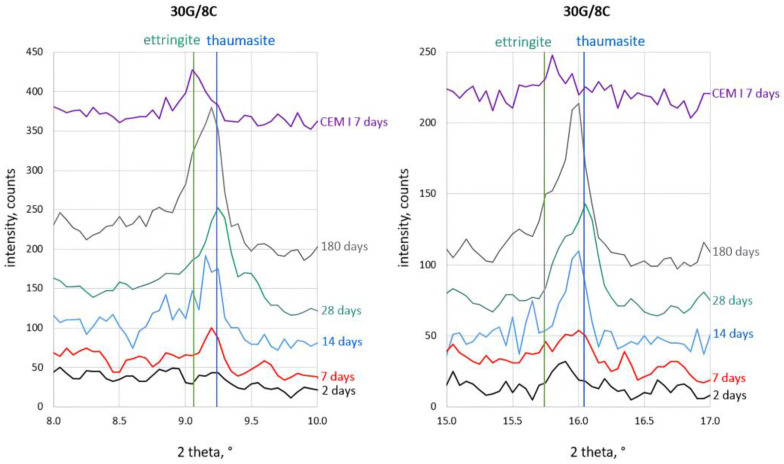
X-ray diffraction patterns of 30G/8C sample cured at increased RH for 2, 7, 14, 28, and 180 days.

**Figure 6:**
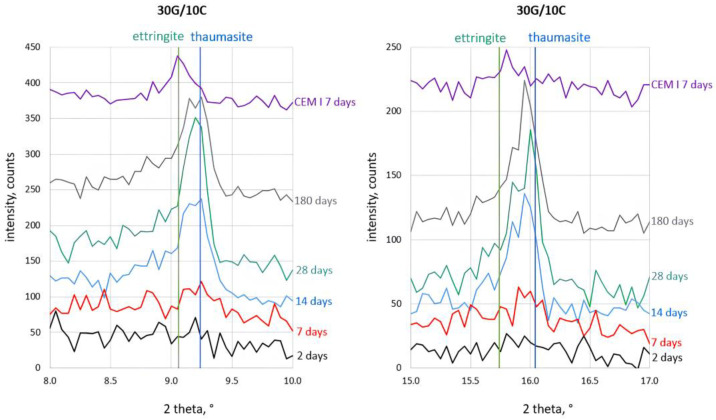
X-ray diffraction patterns of the 30G/10C sample cured at increased RH for 2, 7, 14, 28, and 180 days.

**Figure 7:**
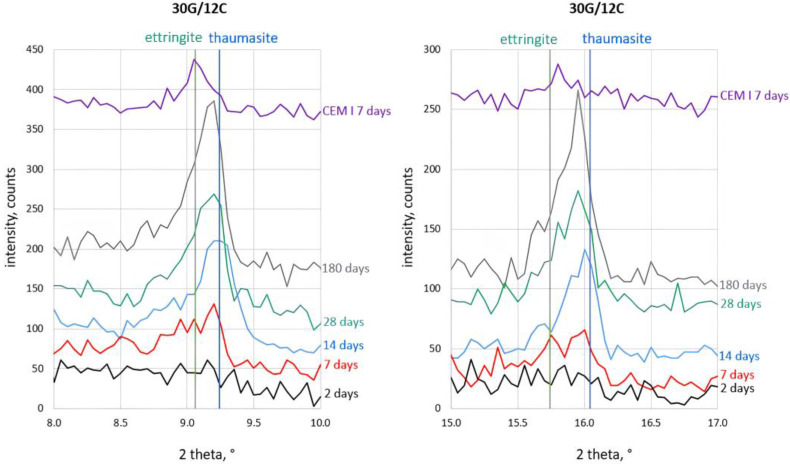
X-ray diffraction patterns of 30G/12C sample cured at increased RH for 2, 7, 14, 28, and 180 days.

The most-interesting observation concerned the process of ettringite and thaumasite formation. The details of the corresponding analysis are shown in Figure 4, Figure 5, Figure 6 and Figure 7. The assignment of the peaks in the XRD patterns to particular phases was performed according to the ICDD database [49]. The XRD pattern of Portland cement (CEM I) paste (w/c = 0.5) after seven days of hydration, which contains ettringite (2θ = 9.06° and 2θ = 15.73°), is included in Figure 4 to support the peak assignment.

The XRD patterns in Figure 4, Figure 5, Figure 6 and Figure 7 show that a small amount of thaumasite formed only after 7 days of curing the samples containing 8 and 12% PC.

Significant changes in all samples’ phase composition occurred after longer curing, including efficient thaumasite formation marked by the increase of their XRD peaks at 2θ = 2.94° and 2θ = 16.06°. The intense peaks of thaumasite became visible in the XRD patterns of all samples after 14 days of curing, and their intensity increased with curing time, reaching the highest values for samples cured for 180 days.

It can be seen, however, that the thaumasite peaks in Figure 4, Figure 5, Figure 6 and Figure 7 are not symmetric or even split. This effect became more visible with increased PC content in the mortars. The asymmetry of the thaumasite peaks may be due to their overlap with the peaks of woodfordite [50], which agrees with Barnett’s results [26,51].

The unambiguous conclusion of whether the samples contained two separate phases of ettringite and thaumasite or a solid solution of these phases is beyond the scope of this study. Nevertheless, the presented results suggested that thaumasite is the main phase in the ettringite–thaumasite system, which influenced the properties of the mortars, including the length changes observed for the studied samples.

One should note that the investigated mortars contained hydrated lime. This is a common additive that acts as a plasticizer, on the one hand, and as a pH modifier, on the other. As can be found in the literature, the presence of calcium hydroxide as an additive promotes thaumasite attack [52]. This may be one of the reasons for the presence of thaumasite in the investigated samples. This also suggests that further tests are necessary to evaluate the role of hydrated lime in the course of thaumasite attack in gypsum–cement mortars since the mentioned work of Bellman et al. [52] was focused on cement-based systems.

The formation of ettringite and thaumasite connects with the Portland cement introduction into the mortars. Doleželová et al. [53] investigated the influence of various supplementary cementitious materials (SCMs) on the properties of gypsum-based mortars with good results. They found that the incorporation of SCMs markedly improved gypsum-based mortars’ performance. Phase analysis performed by the authors did not reveal the presence of thaumasite nor ettringite in the mortars with SCMs. One should remember that the mortars investigated by Doleželová et al. did not contain calcium carbonate.

Direct comparison of the strengths of the mortars investigated in the present study and the mortars described in [53] is difficult. The conclusion was that cement use allowed us to obtain higher mortar strengths compared to SCMs. It is even more significant that the density of the mortars investigated in the present paper was about 1050 kg/m^3^—significantly lower than the mortars with SCMs tested in [53].

### 3.4. Scanning Electron Microscopy and Energy-Dispersive Spectroscopy

**Figure 8:**
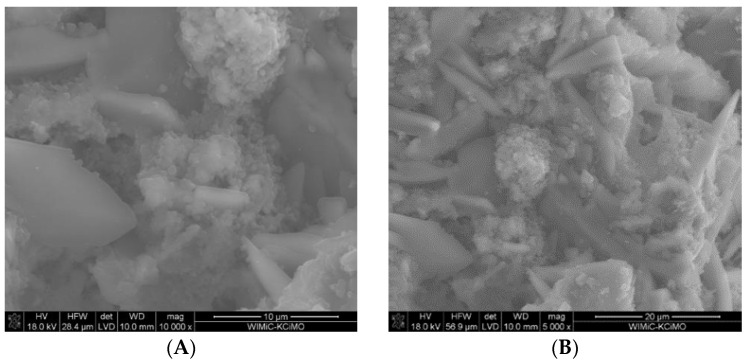
SEM images of the microstructure of the 30G/10C sample cured for 7 days at increased RH. Magnification 10,000 (**A**) and 5000 (**B**).

**Figure 9:**
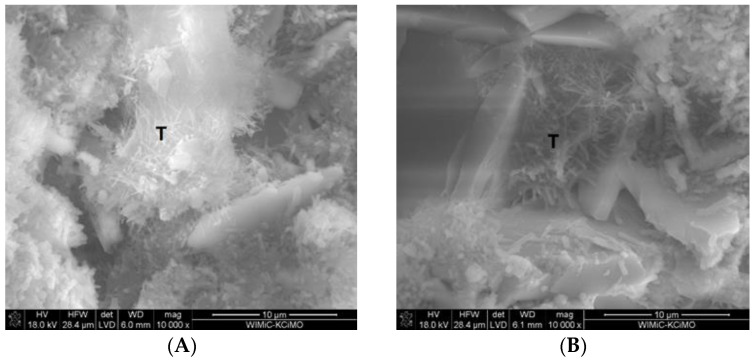
SEM images of the 30G/10C sample cured for 180 days at increased RH; T—thaumasite. Thaumasite crystals formed on the C-S-H phase (**A**) and between gypsum crystals (**B**).

The SEM images of two fractured cross-sections in Figure 8A,B show that the 30G/10C sample cured for 7 days contained numerous gypsum crystals in a loosely packed C-S-H matrix. However, it was found that the microstructure of the mortars changed with increased curing time. The most-significant change comprised the formation of the needle-like structures characteristic of thaumasite or thaumasite–ettringite solid solution. These structures are visible in two exemplary SEM images of fractured cross-sections of the mortars conditioned at increased RH for 180 days (Figure 9A,B).

**Figure 10:**
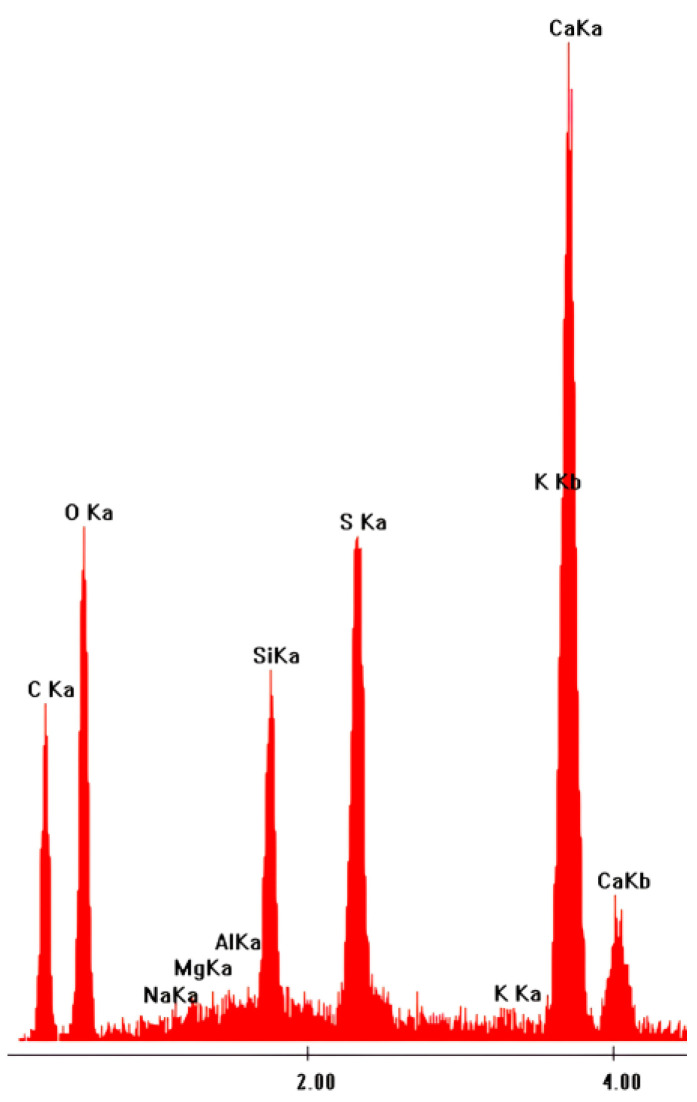
EDS spectrum of thaumasite indicated in Figure 9A,B.

Notably, the presence of thaumasite in the samples was confirmed by the EDS spectrum (Figure 10), which shows the very low signal of Al from the fibrous structures in Figure 9.

## 4. Conclusions

Gypsum–cement mortars containing 30% gypsum and 6 to 12% Portland cement (CEM I 42,5R) addition fulfilled the EN 13279-1:2008 standard’s requirements for gypsum plasters. This result will allow a reduction in the amount of gypsum used in the mortars to 30%, thereby delaying the exhaustion of its supply until it is possible to replace it with another binder.

In addition, it has been shown that the mechanical strength of the studied gypsum–cement mortars containing 30% gypsum increased with the cement content and with a time of curing at high relative humidity (>95%) for the total analysis time of 180 days. This result was attributed to the formation of the calcium silicate hydrate phase (C-S-H), responsible for the increased mechanical strength of the gypsum–cement mortars during mortar curing. It was found that plaster mortar containing even as low as a 6% addition of Portland cement fulfilled the standard’s requirements [7] for flexural and compressive strength after 2 days of curing in increased relative humidity and dry air conditions.

The significant extent of the linear expansion of the samples after six months of curing in the high relative humidity conditions was assigned to the expansive character of the ettringite, thaumasite, and possibly, mixed ettringite–thaumasite phases, whose formation was unambiguously confirmed by the XRD analysis.

The SEM images of the microstructure of the mortar cured for six months at increased relative humidity contained a well-developed C-S-H phase and was tightly packed with the thaumasite and/or mixed ettringite–thaumasite phases. Such a dense structure can account for the observed high durability of the studied plaster mortars.

## Figures and Tables

**Table 1:** Composition of gypsum–cement plasters.

Components	Type of Plaster			
	30G/6C	30G/8C	30G/10C	30G/12C
	%			
Gypsum	30.00	30.00	30.00	30.00
CEM I 42,5R	6.00	8.00	10.00	12.00
Quartz sand (0.1–0.5 mm)	48.59	46.59	44.59	42.59
Limestone powder (0.0–0.2 mm)	10.00	10.00	10.00	10.00
Hydrated lime	2.00	2.00	2.00	2.00
Expanded perlite (0.0–2.0 mm)	3.00	3.00	3.00	3.00
Admixtures Modified methyl cellulose Starch ether Sodium lauryl sulfate L (+) tartaric acid	0.23 0.03 0.02 0.13	0.23 0.03 0.02 0.13	0.23 0.03 0.02 0.13	0.23 0.03 0.02 0.13
Total	100.00	100.00	100.00	100.00

**Table 2:** Expansion of gypsum–cement mortars after 180 days of curing at increased relative humidity (>95%).

Type of Mortar	Expansion	
	mm·m^−1^	%
30G/6C	1.19	0.12
30G/8C	2.54	0.25
30G/10C	6.27	0.63
30G/12C	6.31	0.63

## Data Availability

Not applicable.
